# Macroanatomical Landmarks Featuring Junctions of Major Sulci and Fissures and Scalp Landmarks Based on the International 10–10 System for Analyzing Lateral Cortical Development of Infants

**DOI:** 10.3389/fnins.2017.00394

**Published:** 2017-07-11

**Authors:** Daisuke Tsuzuki, Fumitaka Homae, Gentaro Taga, Hama Watanabe, Mie Matsui, Ippeita Dan

**Affiliations:** ^1^Department of Language Sciences, Tokyo Metropolitan University Tokyo, Japan; ^2^Graduate School of Education, The University of Tokyo Tokyo, Japan; ^3^Applied Cognitive Neuroscience Laboratory, Chuo University Tokyo, Japan; ^4^Research Center for Language, Brain and Genetics, Tokyo Metropolitan University Tokyo, Japan; ^5^Department of Psychology, Graduate School of Medicine and Pharmaceutical Sciences, University of Toyama Toyama, Japan; ^6^Department of Clinical Cognitive Neuroscience, Institute of Liberal Arts and Science, Kanazawa University Kanazawa, Japan

**Keywords:** cortical development, MRI, infancy, 10-20 system, brain template, brain atlas, fNIRS, EEG

## Abstract

The topographic relationships between the macroanatomical structure of the lateral cortex, including sulci and fissures, and anatomical landmarks on the external surface of the head are known to be consistent. This allows the coregistration of EEG electrodes or functional near-infrared spectroscopy over the scalp with underlying cortical regions. However, limited information is available as to whether the topographic relationships are maintained in rapidly developing infants, whose brains and heads exhibit drastic growth. We used MRIs of infants ranging in age from 3 to 22 months old, and identified 20 macroanatomical landmarks, featuring the junctions of major sulci and fissures, as well as cranial landmarks and virtually determined positions of the international 10-20 and 10-10 systems. A Procrustes analysis revealed developmental trends in changes of shape in both the cortex and head. An analysis of Euclidian distances between selected pairs of cortical landmarks at standard stereotactic coordinates showed anterior shifts of the relative positions of the premotor and parietal cortices with age. Finally, cortical landmark positions and their spatial variability were compared with 10-10 landmark positions. The results indicate that variability in the distribution of each macroanatomical landmark was much smaller than the pitch of the 10-10 landmarks. This study demonstrates that the scalp-based 10-10 system serves as a good frame of reference in infants not only for assessing the development of the macroanatomy of the lateral cortical structure, but also for functional studies of cortical development using transcranial modalities such as EEG and fNIRS.

## Introduction

Recent advances in neuroimaging methods have begun to shed light on developmental changes in cortical structures, which could, in turn, serve as a referential framework for describing functional development. Studies using magnetic resonance imaging (MRI) have revealed the development of the macroanatomy of the brain during fetal (Huang et al., 2009; Takahashi et al., 2012), perinatal (Hüppi et al., 1998), postnatal (Gilmore et al., 2012; Oishi et al., 2013; Makropoulos et al., 2016), and childhood and adolescent (Giedd et al., 1999) periods of life. Choosing a frame of reference for 3D images is a fundamental issue in the analysis of individual and developmental variations in structure. While normalization of an individual image to a specific brain image template has been the typical strategy (Evans et al., 2012), this incurs a loss in the variation of structural information that would be of crucial interest for developmental studies. One solution is to look at the brain in reference to the head, and this approach has already manifested in studies of the registration of electroencephalography (EEG) or functional near infrared spectroscopy (fNIRS) sensors attached on the scalp to the underlying lateral cortical structure (for review see Tsuzuki and Dan, 2014). While techniques for the transformation of frame of reference between an MRI of the brain and scalp-based positioning systems, called the 10-20, 10-10, and 10-5 systems, have been established for adults (Okamoto et al., 2004; Jurcak et al., 2007), recent research has begun to present methods for predicting macroanatomical correlates of scalp structures and landmarks on major gyri, sulci, and fissures of the cortex in infants.

There are ongoing efforts to establish large-scale infant MRI datasets (Almli et al., 2007; Altaye et al., 2008). As a reference for the spatial normalization and segmentation of infant MRIs, Altaye et al. (2008) constructed a probabilistic brain template based on MR brain image data from 76 infants ranging in age from 9 to 15 months. Shi et al. (2011) furthered this effort by creating infant brain atlases for neonates to 1- and 2-year-olds based on the MRIs of 95 infants at these three ages. In a functional NIRS imaging study with infants, Watanabe et al. (2013) performed virtual registrations of the 10-20 system and NIRS channels to the neonate automated anatomical labeling (AAL) atlas (Shi et al., 2011) in MNI space (Altaye et al., 2008). On the one hand, virtual registration with adult and neonate brains were shown to be macroanalytically compatible, in agreement with the study by Hill et al. (2010), which showed that a surface-based atlas of the cortex in term infants is similar to that of adults. On the other hand, the normalization of infant brains may be affected by deformations in age-specific patterns of target structures due to the rapid and spatially non-uniform growth of the brain in the first 2 years after birth (Gilmore et al., 2012; Makropoulos et al., 2016), and this may hinder the accuracy of registration. A strategy to overcome this problem is to make age-specific templates of the brain (Sanchez et al., 2012; Richards et al., 2016). Lloyd-Fox et al. (2014) showed that registration of fNIRS probe locations via external head structures to age-appropriate MRI templates (Sanchez et al., 2012) could consistently predict underlying macroanatomical structures in fronto-temporal regions for 4- to 7-month-old infants.

While the aforementioned studies have developed methods for predicting 10-20 standard electrode and/or fNIRS channel locations relative to the underlying macroanatomical structure of the cortex for different ages, few studies have focused on the fundamental issue of how the macroanatomy of the cortex and the skull co-develop and how individual and developmental variations are quantified throughout development. Aldridge et al. (2002) showed that brain morphology of children affected premature closure of cranial sutures differs substantially from that of children without synostosis through an analysis of MRI. Richtsmeier and Flaherty (2013) have reviewed evidence to suggest that the brain and skull co-develop due to shared regulatory influences. Thus, while evaluation of morphology of both of the cortex and the skull is crucial, limited data is available particularly for typical development of infants and children. Matsui et al. (2014) used MRI data for a 12-month-old infant and manually delineated segmented gyri from among the highly visible macroanatomical regions on the lateral cortical surface. Based on external cranial landmarks, these regions were linked to the 10-10 head-surface positioning system. This enabled analysis of the macrostructures of the brain using the skull as a frame of reference. Kabdebon et al. (2014) took two distinct approaches to quantify both the external variability of the 10-20 standard positions over the scalp and the internal variability of the cortical structure in infants ranging in age from 3 to 17 weeks post term. They provided an infant MRI template on which the variability of 10-20 sensor locations with the anatomical variability of major cortical sulci were mapped. The template is based on a representative infant image, as is the case in Tzourio-Mazoyer et al. (2002) and Talairach and Tournoux (1988) for adults and in Matsui et al. (2014) for a 12-month-old infant. Kabdebon et al. found that 10-20 landmarks were mostly robust predictors for underlying macroanatomical structures both for infants and adults, with the exception of some of occipital and temporal electrodes (O1, O2, T5, and T6), which corresponded to lower structures in infants.

In this study, we asked how the brain and head co-develop during the first 2 years of life and whether the head coordinate system provides a good frame of reference not only for the study of the structural development of the brain, but also for the functional study of brain development using EEG and fNIRS in this age period. Thus, we sought a multi-facetted solution for this problem. We focused on several macroanatomically distinct cortical structures on the lateral cortical surfaces of infants between the ages of 3 and 22 months old. These structures were mainly selected at junctions of major sulci and fissures, which can be detected even in low-contrast MR images. Next, we examined topographic changes of the locations of these macroanatomically distinct structures during infant development. We attempted two different approaches for this examination. First, we performed a Procrustes analysis (Bookstein, 1996; Zelditch et al., 2012) to find any holistic trends in relative distribution among cortical macroanatomical landmarks in the course of infant development. The Procrustes registration to minimize and normalize spatial differences among landmarks and the principal component analysis (PCA) to analyze residuals have been used to reveal variations of shapes of the cortex (Free et al., 2001; Weinberg et al., 2009; Bruner et al., 2017). Second, we described the cortical landmark positions in reference to commonly used referential landmarks, namely the anterior and posterior commissures to set the origin to their mid point, the y-axis through the AC-PC line, and the z-axis through the midline (Talairach and Tournoux, 1988). Using this coordinate system, the cortical landmark positions were assessed in reference to others across developmental stages. Then, these landmark positions and their spatial variability were compared with 10-10 landmark positions. We assumed that there would be some developmental trends in the distribution of the lateral cortical landmarks representing the temporally and spatially uneven development of gyri. The fundamental purpose of the current study was to find whether such developmental changes are smaller or greater than the lateral cortical regions defined by the 10-10 system when both the brain and head grow rapidly. In other word, we assessed whether the 10-10 system can serve as a robust predictor of macroanatomy estimated from the scalp of infants ranging in age from birth to 2 years in developmental studies using transcranial modalities such as EEG and fNIRS.

## Methods

### Image acquisition

The MRI data of normally developing infants were obtained from the MRI data set previously reported (Tanaka et al., 2012; Uematsu et al., 2012). Original participants included 114 healthy and normally developing Japanese (60 males and 54 females) from 1 month to 25 years old (mean age in months ± S.D.: 106.00 ± 83.15). Their entire study was approved by the Research and Ethics Committee at the University of Toyama. After the purpose and all study procedures were fully explained, written informed consent was obtained from all adult research participants and from the parents/legal guardians of all non-adult research participants. They acquired MRI data as follows. T1-weighted axial images with 1.0 mm thickness were obtained on a 1.5T Magnetom Vision scanner (Siemens, Erlangen, Germany) for each participant while asleep, using the fast low angle shot gradient refocused three-dimensional sequence with the following parameters: echo time (TE) = 6 ms, repetition time (TR) = 35 ms, flip angle = 35°, nex = 1, field of view = 256 mm, and matrix size = 256 × 256. Each entire scan was completed in 15 min. From the resulting MRI pool, we chose the 16 youngest participants, from 3 to 22 months (mean: 11.06 months; 10 males and 6 females), with sufficient MR image quality, which can allow us to differentiate gray matter from cerebrospinal fluid by visual inspection to determine major sulci and junctions. It is challenging to perform automatic segmentation of young infants' MR images into white matter, gray matter and cerebrospinal fluid due to the low spatial resolution, severe partial volume effect, high image noise, and dynamic myelination and maturation processes (Wang et al., 2014). We previously delineated sulci and gyri of a 12-month-old infant in this infant group (Matsui et al., 2014).

### Image preprocessing

For the present study, acquired MRI data were aligned to the anterior commissure (AC) and posterior commissure (PC), with the AC-PC midpoint providing the origin, the AC-PC line forming the y-axis, and a midline forming the z-axis. The x-axis ran left to right. Skull stripping was performed using the automated brain extraction tool (BET; Smith, 2002) provided as a part of the FMRIB Software Library (FSL, http://fsl.fmrib.ox.ac.uk/fsl/fslwiki/; Smith et al., 2004). Fractional intensity threshold was determined for all MRI data.

### Anatomical landmarks

**Figure 1:**
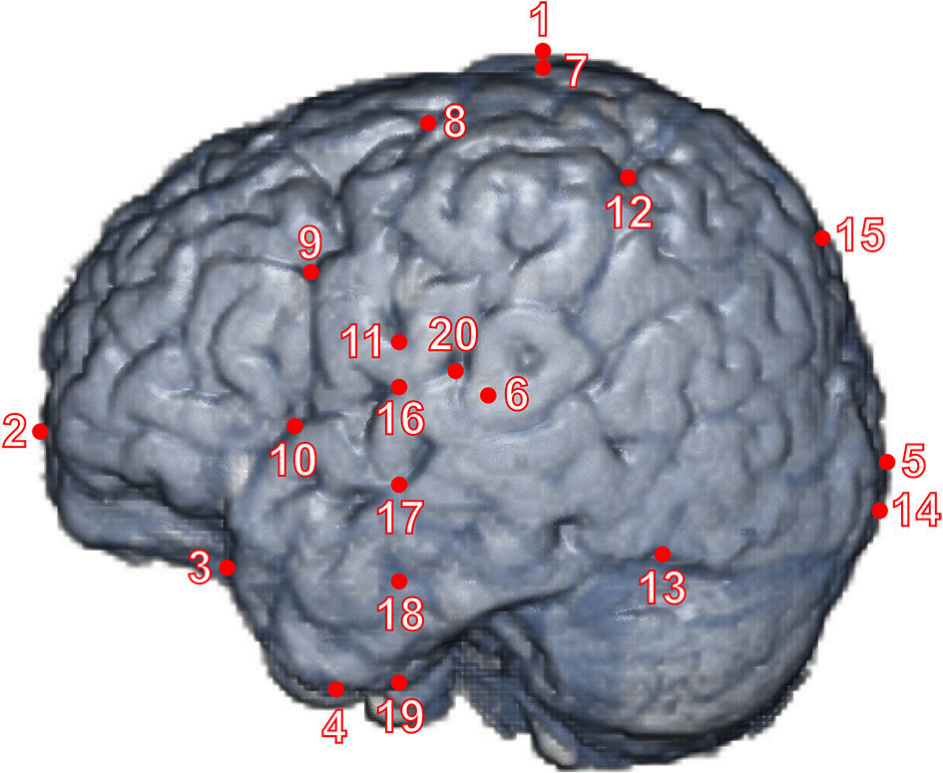
The landmarks on a brain surface of a representative infant. We defined 20 landmarks on each hemisphere of the cerebral cortex. The red dots (from No. 1–6) are extreme points, including poles, in antero-posterior, ventro-dorsal, and left-right directions. The red dots (from No. 7–20) are cortical landmarks (see Section Materials and Methods). We delineated all major sulci of a 12-month-old infant in our previous study (Matsui et al., 2014), and the landmarks are rendered on the left surface of the infant brain for presentation purposes.

Using the MRIcron software package (Rorden et al., 2007), we registered 20 macroanatomical cortical landmarks as described below for each cerebral hemisphere in three-dimensional (3D) MRI data (Figure 1). They represent the intersection of major sulci and fissures or their termination on the lateral surface of the cortex. Pre-auricular points (left: AL, right: AR), which were defined at the anterior roots of the tragi, as well as the Nasion (Nz) and Inion (Iz) were determined on the MRI to obtain international 10-20 and 10-10 positions of electrodes. First, two primary raters (ID, FH) independently assessed the lateral surface structures and MRI slices, and, upon discussion, determined target landmarks. Second, at least two secondary raters independently marked the landmarks for each infant MRI and recorded the 3D coordinates. Finally, the primary raters evaluated the resulting sets of coordinates, which showed no major mismatches among raters, and adopted the mode or mean values of the coordinates as the coordinates of the landmarks. The inter-rater difference in 3D coordinate distance averaged over 40 landmarks and 16 individuals was 4.87 ± 6.20 mm. The inter-rater agreement was obtained at 86.25% of 40 landmarks on average among individuals under the precision of 11.07 mm, which was one *SD* above the mean of the inter-rater difference in 3D coordinate distance. The identification method of sulci was described in our previous study (Matsui et al., 2014). The specific definitions for macroanatomical landmarks are as follows:

1. Vertex: We determined the highest axial slice in which the parietal cortex appeared, and recorded the coordinates of the center-of-gravity of the parietal cortex in that axial slice.
2. Frontal pole: We determined the coronal slice in which the frontal cortex appeared, and recorded the coordinates of the center-of-gravity of the frontal cortex in that coronal slice.
3. Temporal pole: We determined the most anterior coronal slice in which the temporal cortex appeared, and recorded the coordinates of the center-of-gravity of the temporal cortex in that coronal slice.
4. Base of the brain: We determined the most ventral axial slice in which the temporal cortex appeared, and recorded the coordinates of the center-of-gravity of the temporal cortex in that axial slice.
5. Occipital pole: We determined the most posterior coronal slice in which the occipital cortex appeared, and recorded the coordinates of the center-of-gravity of the occipital cortex in that coronal slice.
6. Leftmost and rightmost points: We determined the leftmost and rightmost sagittal slices in which the temporal cortex appeared, and recorded the coordinates of the center-of-gravity of the temporal cortex in the sagittal slice for each side.
7. Upper edge of the central sulcus: We identified the central sulcus, and found the most dorsal axial slice where the posterior bank of the central sulcus could be determined. We recorded the most medial coordinates of the posterior bank on that axial slice.
8. Junction of the superior frontal sulcus and the precentral sulcus: We identified the superior frontal sulcus and the precentral sulcus, and found the junction of these sulci that appears on the axial slices. Hereafter, we refer to an intersection of two sulci as a “junction” according to Destrieux et al. (2010). We also determined the neighboring point of the junction on the lateral surface using the coronal slice.
9. Junction of the inferior frontal sulcus and the precentral sulcus: We identified the inferior frontal sulcus, and found the coronal and sagittal slices where the junction of this sulcus and the precentral sulcus appeared. We also determined the neighboring point of the junction on the lateral surface using the axial and coronal slices.
10. Root of the ascending ramus of the Sylvian fissure: We identified the Sylvian fissure and the ascending ramus of the Sylvian fissure. We found the root of the ramus on the most lateral (sagittal) slice. The neighboring point of this landmark on the lateral surface was also determined using the axial and coronal slices.
11. The inferior termination of the central sulcus (CS tip): We determined the inferior termination of the central sulcus that appears on the axial slice and recorded the coordinates of the most lateral point. The central sulcus sometimes connects with the Sylvian fissure. In this case, the junction of the central sulcus and the Sylvian fissure was considered the inferior termination of the central sulcus.
12. Junction of the posterior central sulcus and the intraparietal sulcus: We identified the posterior central sulcus and the intraparietal sulcus, and found the junction of these sulci on the axial and sagittal slices. We also determined the neighboring point of the junction on the lateral surface using the coronal slice.
13. Preoccipital notch: We identified the axial and sagittal slices where the preoccipital notch was most visible. We sometimes used a surface reconstruction of the MRI data to confirm the position. The most ventral and lateral coordinates were recorded.
14. Calcarine fissure: We identified the calcarine fissure using the axial and sagittal slices, and determined the most medial point where the fissure appeared on the lateral surface around the occipital pole.
15. Parietooccipital sulcus: We identified the parietooccipital sulcus on the sagittal slice and recorded the coordinates of the most dorsomedial point of the sulcus, which was observed on axial and sagittal slices.
16. Sylvian fissure on the coronal slice of CS tip: We determined the most lateral point of the Sylvian fissure on the coronal slice of the y coordinate of landmark
17. Superior temporal sulcus on the coronal slice of CS tip: We identified the superior temporal sulcus and determined the most lateral point of the sulcus on the coronal slice of the y coordinate of landmark 11.
18. Inferior temporal sulcus on the coronal slice of CS tip: We identified the inferior temporal sulcus, and determined the most lateral point of the sulcus on the coronal slice of the y coordinate of landmark 11.
19. The most ventral point of the lateral temporal cortex on the coronal slice of CS tip: On the coronal slice of the y coordinate of landmark 11, we determined the most ventral point of the lateral temporal cortex (This point was only used for analyzing relationship between 10-10 positions and macroanatomical landmarks).
20. Root of the posterior ascending branch of the Sylvian fissure: We determined the root of the posterior ascending branch of the Sylvian fissure on the sagittal slice, where the inclination of the Sylvian fissure changed drastically, and recorded its coordinates and the coordinates of the neighboring point on the lateral surface using axial and coronal slices.

In a previous study, 12 landmarks distributed over the cortical surface were used to assess shape difference of the normal adult brain (Free et al., 2001). They chose the landmarks for their reliability of identification in anatomical term and with respect to the limitations of the MRI data and the segmentation process. Of the 12 landmarks, 3 on the medial surface were not used in the present study. Of the remaining 9 landmarks on the lateral surface, 8 correspond to the ones we used in the present study. In addition to the above landmarks, we used 2 landmarks to evaluate the changes in the parietal and occipital cortices on the lateral surface. Chollet et al. (2014) developed protocols for cortical and subcortical landmarks to quantify changes in the brain shape. By using 10 structural images of 12 years old boys, they determined 3 landmarks for poles of the cerebrum, 8 landmarks on the lateral surface of the cerebral cortex, and 18 landmarks on the subcortex. We used all 3 landmarks for poles and 2 additional landmarks in order to measure the height and width of the cerebrum. Of 8 landmarks on the lateral surface, 5 landmarks were used in the present study. A landmark on the ascending ramus of the sylvian fissure was employed in both previous and present studies, though the different terminations (i.e., superior or inferior ends) were selected. In addition to the 6 landmarks on the lateral surface, we determined 4 landmarks in the parietal and occipital cortices as mentioned above. Thus, the landmarks of the present study cover wider regions of the lateral surface of the cortex to be used for shape analysis of the developing brain.

### Procrustes analysis

To analyze holistic variations in the shapes of the cortex and scalp using the Procrustes method (Bookstein, 1996; Zelditch et al., 2012), landmark positions were chosen from each cortex and head of the 16 participants. For the cortex, 10 landmarks (numbers 7–15, and 20) defined by fissures, sulci, and their junctions for each of the hemispheres were chosen from the aforementioned 20 landmark points. Landmarks 1–6 were not used for the analysis because they were points defined by maximum or minimum coordinate values, but not by distinct macroanatomical cortical structures. Landmarks 16–19 were not used for the analysis because their positions were dependent on the position of landmark 11 located on the same coronal slice. For the head, 25 landmarks from among the 10-20 positions were used for the analysis. Using manually determined positions for AL, AR, Nz, and Iz on the MR image of the head, the virtual 10-20 measurement method (Jurcak et al., 2007) was applied to determine other 10-20 positions. Procrustes analysis was performed using the software MorphoJ (Klingenberg, 2011). First, centroid size was computed as the square root of the sum of the squared distances of the landmarks from the centroid position. All configurations were then scaled to a standard size based on centroid size. Second, all configurations were translated so that their centroid positions corresponded with coordinate origins. Finally, configurations were rotated to bring all configurations into an optimal orientation in which the sum of the squared deviations between corresponding landmarks was minimal. The resulting landmark coordinates are called Procrustes shape coordinates. Coordinate transformation was performed for each of the cortex and head landmarks. To examine the main features of shape variation, a PCA was performed. Shape changes along the principal components were visualized in the form of vectors for each landmark. To assess the developmental changes in shape, we further examined if principal component scores of individuals can regress on age (Zelditch et al., 2012).

### Calculation of topological relationships among macroanatomical landmarks

Basically, Euclidian distances between given macroanatomical landmarks were used to assess their topological relationship (indicated as “direct distance” in Table 1). Ratios between a set of two given landmark distances were calculated. Specific macroanatomical landmarks used for analyses are depicted in Figure 1. When junctions of sulci and fissures were detected on slice images, they tended to be located beneath the lateral cortical surface. To avoid variability in depth direction, their locations were adjusted to the cortical surface. Macroanatomical structures around the medial cortical surface were projected onto the sagittal plane for analysis. To analyze the height of macroanatomical structures, they were projected onto the coronal plane. Correlation analyses were performed between a given ratio for topological relationship and age of infant (months). All calculations were performed using a numerical processing software package, Matlab 2007b (MathWorks).

**Table 1:** Topological relationship among macroanatomical cortical landmarks using Euclidian distances.

**Landmark**	**R/L**	**Definition**	**Mode**	***r***	***P***	**Interpretation**
7. Upper end of the central sulcus	L	(2-7)/(7-5)	Projected to the sagittal plane	−0.034	0.900	Unchanged along anterior-posterior axis
	R			−0.018	0.949	
8. Junction of the superior frontal sulcus and the precentral sulcus	L	(2-8)/(8-5)	Direct distance	−0.620	0.010	Relative position around the upper precentral sulcus shifts anteriorly
	R			−0.570	0.021	
9. Junction of the inferior frontal sulcus and the precentral sulcus	L	(2-9)/(9-5)	Direct distance	−0.725	0.002	Relative position around the lower precentral sulcus shifts anteriorly
	R			−0.742	0.001	
10. Root of the ascending ramus of the Sylvian fissure	L	(2-10)/(10-5)	Direct distance	−0.594	0.015	Relative position of the ascending rami of the Sylvian fissures shifts anteriorly
	R			−0.555	0.026	
16. Lower (imaginary) root of the central sulcus	L	(2-16)/(16-5)	Direct distance	−0.579	0.019	Significant anterior shift
	R			−0.562	0.023	
12. Junction of the posterior central sulcus and the intraparietal sulcus	L	(2-12)/(12-5)	Direct distance	−0.677	0.004	Relative position of the junction of the posterior central and the intraparietal sulci shifts anteriorly
	R			−0.705	0.002	
15. Parieto-occipital sulcus	L	(2-15)/(15-5)	Projected to the sagittal plane	−0.447	0.082	Moderate tendency of anterior shift of relative position of the parieto-occipital sulcus
	R			−0.420	0.105	
20. Root of the ascending brunch of the posterior Sylvian fissure	L	(2-20)/(20-5)	Direct distance	−0.397	0.127	Moderate tendency of anterior shift of relative position around the supramarginal gyrus
	R			−0.417	0.108	
13. Preoccipital notch	L	(2-13)/(13-5)	Direct distance	−0.177	0.512	Relative position of the preoccipital notch remain unchanged along anterior-posterior axis
	R			−0.253	0.345	
14. Calcarine fissure height	L	(1-14)/(14-4)	Projected to the coronal plane	−0.768	0.000	Significant dorsal shift
	R			−0.659	0.006	
20. Sylvian fissure height	L	(1-20)/(20-4)	Projected to the coronal plane	0.189	0.482	Unchanged
	R			0.284	0.286	
17. Superior temporal sulcus height	L	(1-17)/(17-4)	Projected to the coronal plane	−0.301	0.258	Unchanged
	R			0.356	0.177	
18. Inferior temporal sulcus height	L	(1-18)/(18-4)	Projected to the coronal plane	0.327	0.216	Unchanged in the left, while significant lowering in the right hemisphere
	R			0.524	0.037	

*Landmark numbers are as described in the main text and Figure 1. Definition represents the Euclidian distance ratio used. Mode indicates how distances are calculated. r represents correlation coefficients. P represents probability of the correlation. R represents right, and L represents left*.

### Virtual setting of 10-10 landmarks on infant scalps

As a relative head-surface-based positioning system, we utilized the international 10-10 system (Chatrian et al., 1985), which has served as a robust predictor of macroanatomy on the lateral cortical surface (Okamoto and Dan, 2005; Kabdebon et al., 2014; Matsui et al., 2014). Briefly, we extracted the head surface from the infant MRI data set using an in-house software program that applies a 3D edge detection algorithm. Then, the 10-10 landmark positions were determined according to the automatic unambiguously illustrated (UI) 10-10 method (Jurcak et al., 2007). We used four cranial landmarks, Nz, AL, AR, and Iz, as initial reference points to apply the automatic UI 10-10 method to each infant scalp.

### Transformation of macroanatomical landmarks via 10-10 landmarks

**Figure 2:**
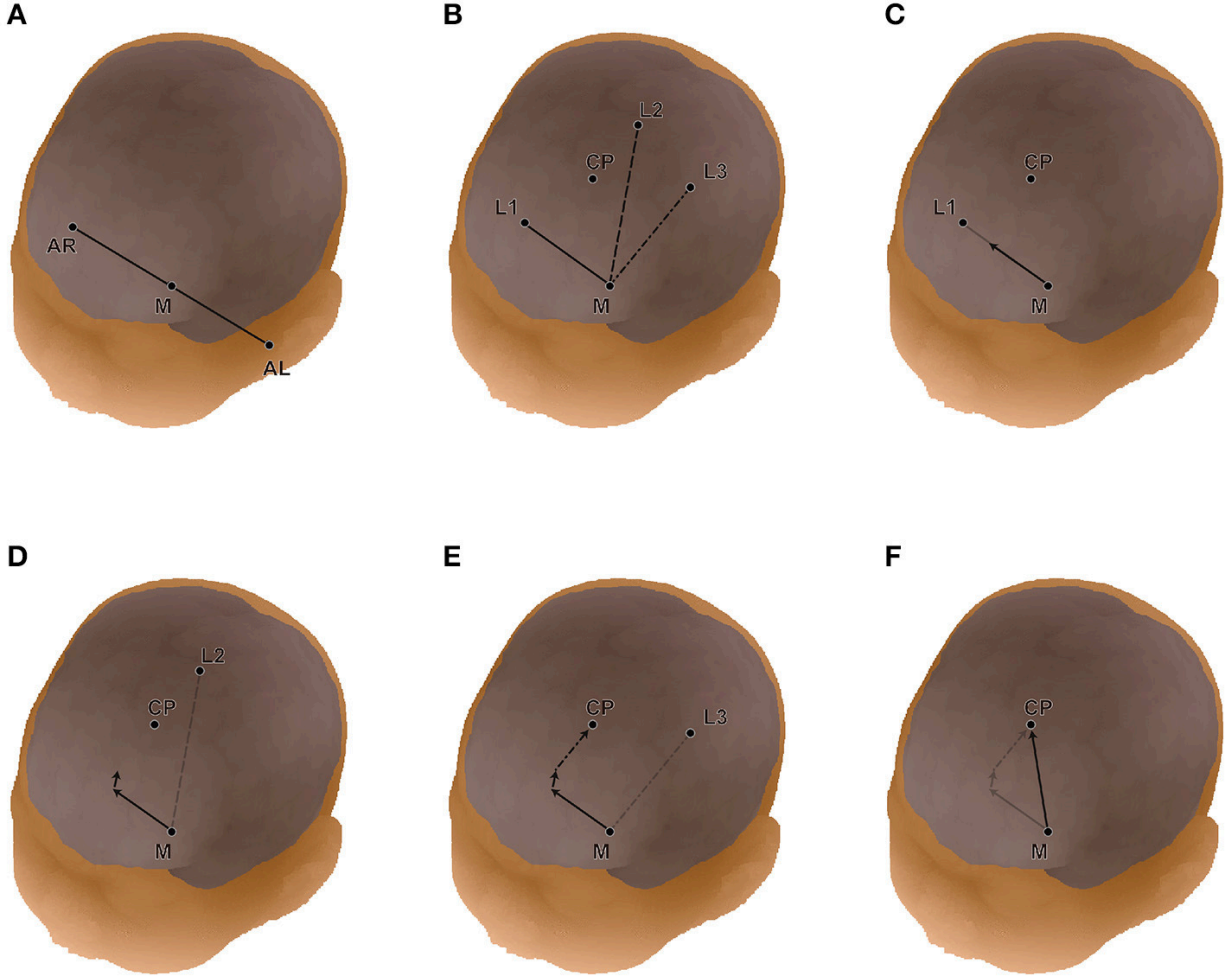
Detailed expression of linear algebraic representations of cortical anatomy with cranial landmarks. **(A)** The midpoint (M) of AL-AR is determined. **(B)** The three landmarks (L1, L2, and L3) on the head surface neighboring the cortical position (CP) are chosen automatically. In this study, we used the international 10-10 system for the cranial landmark set. **(C)** Three coefficients (a1, a2, a3) are obtained for vectors from M to L1**(C)**, M to L2 **(D)**, and M to L4 **(E)**. **(F)** Finally, the vector from M to CP is re-expressed in reference to the four distinct points (or the three vectors).

We analyzed the spatial variability of the macroanatomical cortical landmarks against the 10-10 landmarks. In infant head space, the relative location of a given point on the cortex (CP) can be expressed as a composition of vectors that refers to neighboring standard points of the infant's head surface such as the 10-10 landmarks (Okamoto and Dan, 2005). Note that any macroanatomical landmark can be a CP. As one of four distinct points, we used the midpoint, M, of the AR-AL vector (Figure 2). We designated M for a given subject as M_s_. The three neighboring 10-10 landmarks of the CP were selected as the other three points, and designated as L1, L2, and L3. For a given subject, these points were designated as CP_s_, L1_s_, L2_s_, and L3_s_. The position of CP_s_ was expressed using the three vectors from M_s_. We obtain three coefficients, a1_s_, a2_s_, and a3_s_, for each of the three vectors. That is,

(1) $$\vec{M_{s}CP_{s}}=\;a1_{s}\cdot \;\vec{M_{s}L1_{s}}+\;a2_{s}\cdot \;\vec{M_{s}L2_{s}}+a3_{s}\cdot \;\vec{M_{s}L3_{s}}$$

Then, we obtained the corresponding locations of the selected macroanatomical landmarks on the 12-month-old infant template (Matsui et al., 2014). We assigned the three coefficients to the three corresponding vectors in the template's space. When these vectors are designated with a suffix, small t, the corresponding cortical point on the template space, CP_t_ is obtained as follows:

(2) $${\vec{M_{t}CP}}_{t}=\;a1_{s}\cdot \;\vec{M_{t}L1_{t}}+\;a2_{s}\cdot \;\vec{M_{t}L2_{t}}+a3_{s}\cdot \vec{M_{t}L3_{t}}$$

Thus, the selected macroanatomical cortical landmarks of an infant brain were transferred to the 12-month-old infant atlas space. However, since these points are located around the cortical surface of the 12-month-old infant brain, they were back-projected onto the scalp by linearly enlarging the three coefficients. In this way, all the macroanatomical cortical landmarks of the 15 infants were transformed to the scalp of the 12-month-old infant template in reference to the 10-10 landmarks.

## Results

### Overall tendencies

**Figure 3:**
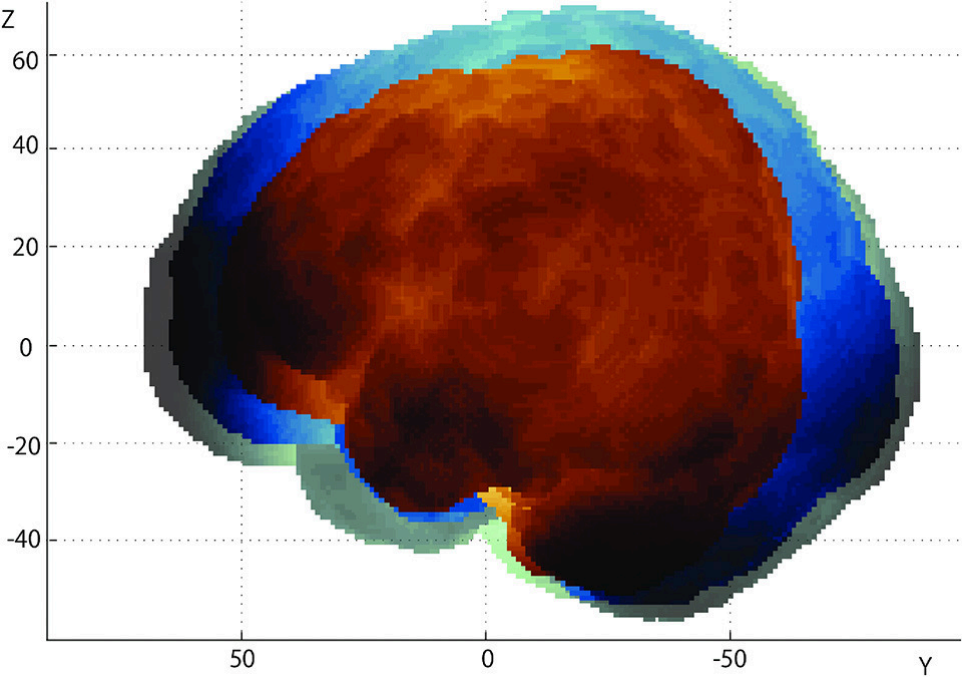
Growth of the infant brain. We have set the AC-PC midpoint of each brain as the origin of the 3-D coordinates, and overlaid the left lateral views of three brains of 3- (brown), 10- (blue), and 18-month-old (green) infants. Note the elongation of the brain in the anterior-posterior direction (y-axis).

The infant brains used in the current study were aligned to a coordinate system with the AC-PC midpoint as the origin (Figure 3). For descriptive purposes, three brains from 3-, 10-, and 18-month-old infants were selected. Brain sizes significantly increased as infants developed: age-dependent growth was most prominent for length (L: *r* = 0.846, *P* = 0.000; R: *r* = 0.867, *P* = 0.000), followed by height (L: *r* = 0.773, *P* = 0.000; R: *r* = 0.580, *P* = 0.019), whereas changes of width exhibited only a moderate correlation with age (L: *r* = 0.465, *P* = 0.069; R: *r* = 0.512, *P* = 0.042).

### Procrustes analysis

**Figure 4:**
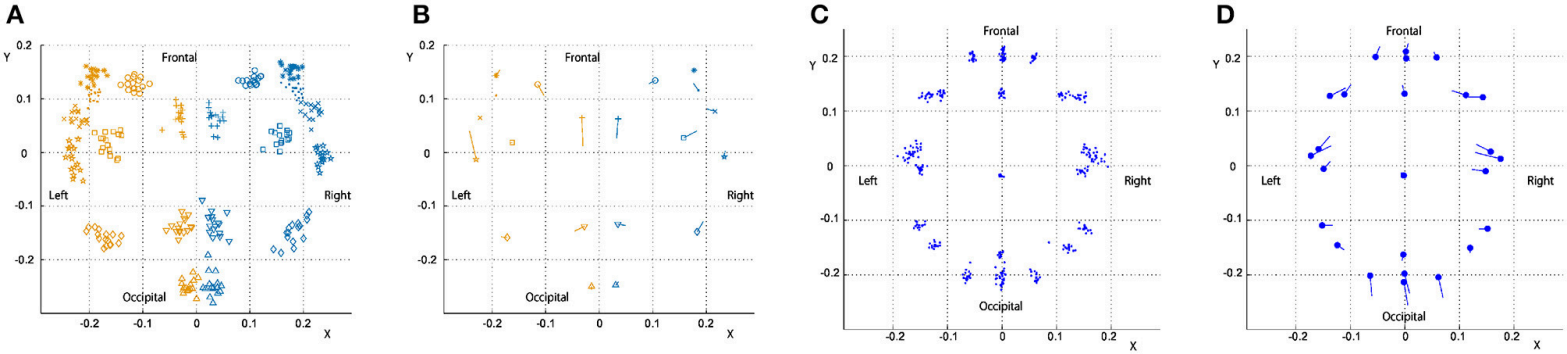
Procrustes analysis of the cortical and head landmarks. Positions of landmarks of individuals and averaged positions of landmarks with the direction of the first principal component of variations are separately shown for the cortex and the head superimposed on the Procrustes coordinates. **(A)** Cortex (individual positions). **(B)** Cortex (averaged positions with the principal component). **(C)** Head (individual positions). **(D)** Head (averaged positions with the principal component).

**Figure 5:**
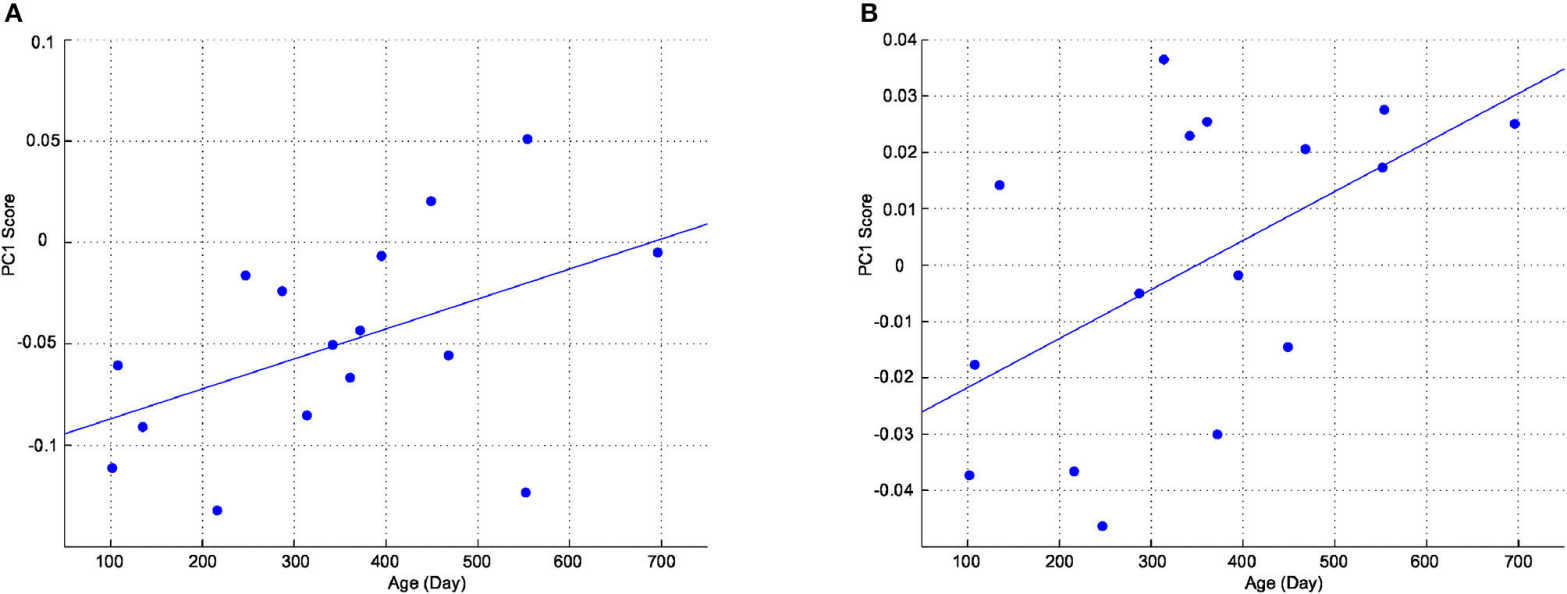
First principal component scores as a function of age. **(A)** Cortex. **(B)** Head.

To examine changes in the shape of the cortex and head with age, positions of cortical, and head landmarks were analyzed using Procrustes analysis. Figure 4A shows landmark positions of the cortices of all participants superimposed on the Procrustes coordinates. A PCA was performed to the individual shape variables of the cortices on the Procrustes coordinates. The first four principal components account for 20.6, 15.6, 11.6, and 10.4% of the total sample variation, respectively. As shown in Figure 4B, left and right landmark 7 (top of the central sulcus) and left landmark 20 (root of the posterior ascending branch of the Sylvian fissure) had greater shape variations along the first principal component. Figure 4C shows landmark positions of the heads of all participants superimposed on the Procrustes coordinates. A PCA was performed to the individual shape variables of the heads on the Procrustes coordinates. The first four principal components account for 31.3, 24.2, 16.4, and 12.3% of the total sample variation, respectively. As shown in Figure 4D, points O1, O2, Oz, T3, T4, F7, and F8 exhibited greater variation along the first principal component. The first principal component scores of the cortical and head landmarks had a correlation with age (*r* = 0.48; *P* < 0.05, and *r* = 0.54; *P* < 0.05, respectively), as shown in Figure 5.

### Topological relationship among macroanatomical landmarks using euclidian distance

The results of analyses of the topological relationship among macroanatomical landmarks on the lateral cortical surface of infant brains are summarized in Table 1. We found that the relative positions of the macroanatomical structures on the premotor cortex as well as the parietal cortex shift anteriorly with age.

### Relationship between 10-10 positions and macroanatomical landmarks

**Figure 6:**
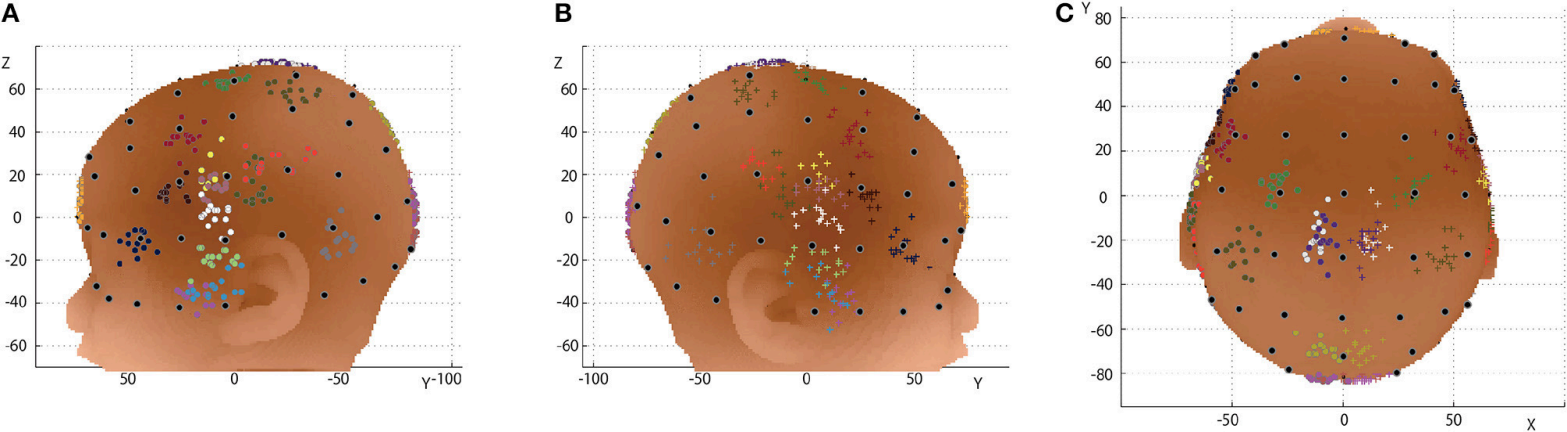
Relationship between 10-10 positions and macroanatomical landmarks. 10-10 landmarks (black dots) are depicted on the 12-month-old infant scalp template. The macroanatomical cortical landmarks from 14 infants, transformed using three neighboring 10-10 landmarks and projected onto the scalp, are visualized in different colors: 1, sky gray; 2, orange; 3, navy blue; 4, cherry pink; 5, magenta; 6, grass green; 7, violet; 8, malachite green; 9, brown; 10, wine red; 11, yellow; 12, olive green; 13, silver gray; 14, terracotta; 15, khaki; 16, burnt sienna; 17, white; 18, cobalt green; 19, blue and 20, red, where the numbers indicate the macroanatomical structures depicted in Table 1. **(A)** Left lateral view. **(B)** Right lateral view. **(C)** Top view.

**Figure 7:**
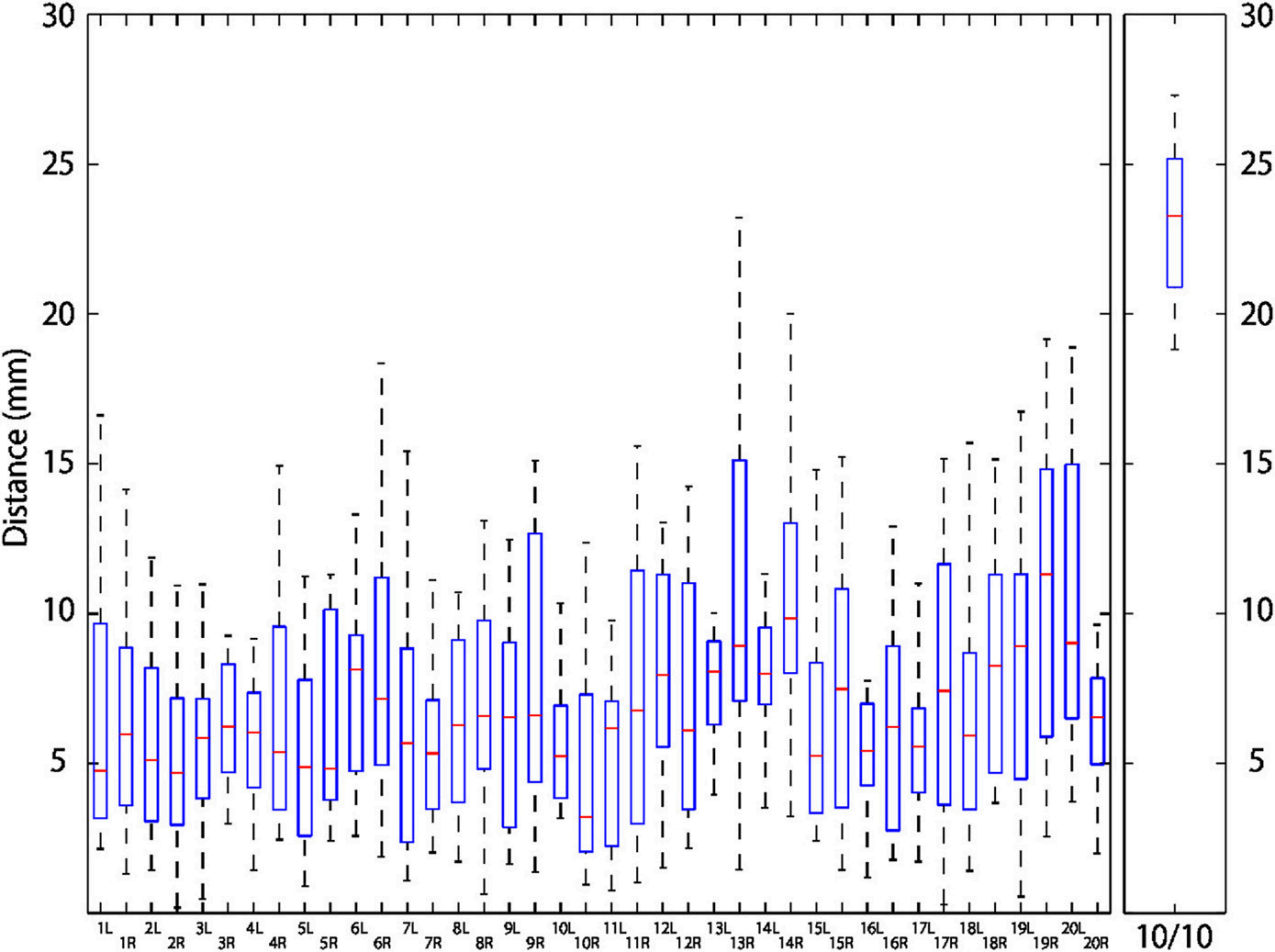
Distribution of macroanatomical cortical landmarks projected on the scalp. Left and right macroanatomical cortical landmarks of 14 infants were transformed and projected onto the 12-month-old infant scalp template. Distances from their centroids are depicted using box-and-whisker plots where the horizontal red bars indicate the median, boxes represent upper and lower quartiles, and the upper and lower whiskers correspond to the smallest distance above and the largest distance below 1.5 inter-quartile range from the upper and lower quartiles. In the rightmost panel, distribution of the nearest distance from each 10-10 landmark is also depicted in a box-and-whisker plot as described above.

Macroanatomical landmarks of 14 infants were transformed to the scalp surface of a 12-month-old infant based on their relative relation with three neighboring 10-10 positions, and were depicted in reference to the 10-10 positions (Figures 6A–C). The data of two infants were not used for this analysis because the field of view of the MRIs was insufficient to accurately calculate the virtual 10-10 positions. As depicted in Figure 7, the median distance from the centroid of each macroanatomical landmark was <12 mm, and the upper quartile remained below 15 mm (note that the length of the scalp is about 15 cm). For all the 10-10 positions, the distance to the nearest 10-10 position were calculated. As shown in Figure 7, the median distance was 23 mm, with upper and lower quartiles of 25 and 21 mm, respectively. Thus, variability in the distribution of each macroanatomical landmark was much lower than the pitch of the 10-10 landmarks.

**Figure 8:**
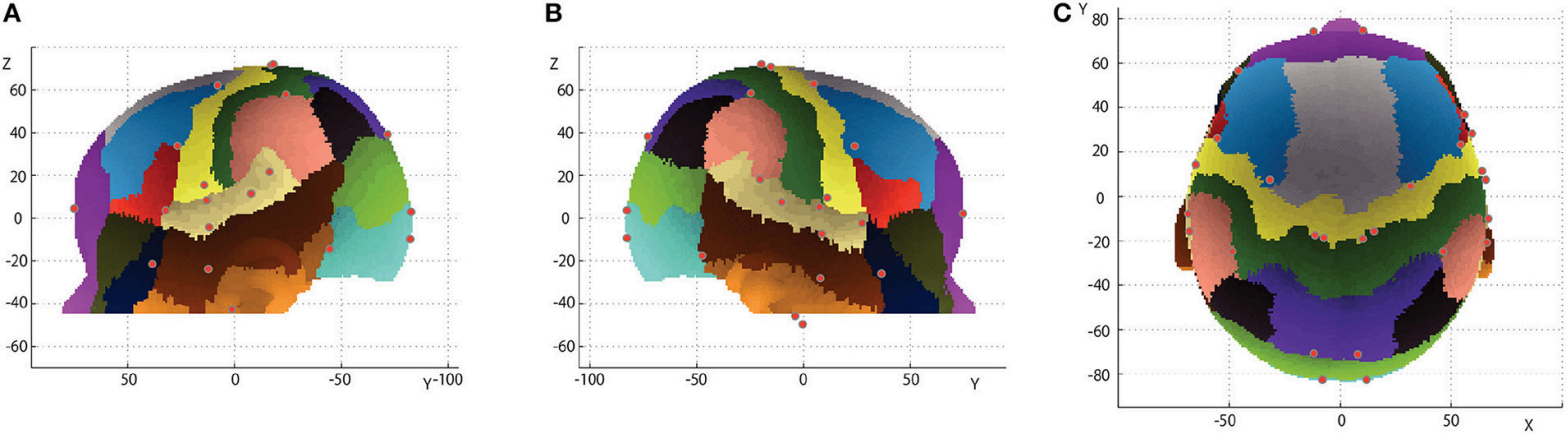
Inter-subject centroids of the macroanatomical cortical landmarks on delineated gyri of the 12-month-old template. The delineated cortical gyri are projected onto the scalp (Matsui et al., 2014). Red dots indicate cortical landmarks defined with maximum or minimum coordinate values or macroanatomical cortical landmarks (No. 1–20 in Figure 1). **(A)** Left lateral view. **(B)** Right lateral view. **(C)** Top view.

To confirm the robustness of the macroanatomical cortical landmarks, centroids for each landmark were projected onto the 12-month-old scalp template with delineated gyri as described in Matsui et al. (2014). As shown in Figure 8, there were no major discrepancies between the locations of the centroids and the macroanatomical template-specific landmarks.

## Discussion

### Overview

The present study describes developmental changes in the topographical relationships among macroanatomical landmarks on the lateral cortical surface of developing infants between the ages of 3 and 22 months. The Procrustes analysis, focusing on holistic trends in relative distribution among the macroanatomical cortical landmarks, revealed a principal component represented by a bilateral posterior shift of the upper region around the central sulci and a left-lateral anterior shift of the region around the posterior edge of the Sylvian fissure as infants develop. In addition, the Procrustes analysis performed with the presence of 10-20 landmarks on the scalp revealed principal component represented by an elongation of the scalp landmark positions along the anterior-posterior axis. On the other hand, analyses of the topological relationships among macroanatomical landmarks from stereotactic coordinates indicated several developmental changes in relative positions among the cortical landmarks including the root of the central sulci, the root of the ascending branches of the posterior Sylvian fissures, the root of the ascending rami of the Sylvian fissures, the posterior root of the inferior and superior frontal sulci and the calcarine fissures. Despite the presence of developmental changes in relative locations of the cortical landmarks when expressed in stereotactic coordinates, their variability was rather small compared to cortical regions defined by the international 10-10 system. In the following discussion, we will examine the importance of these findings from developmental and technical points of view, and also present perspectives on how these findings can be practically implemented in fNIRS and EEG studies on infants.

### Procrustes analysis

The sample size of 16 participants ranging in age from 3 to 22 months in the present study was not enough to perform a group analysis. However, significant regressions of individual shape variables of cortical and scalp landmarks on age suggest that the present analysis captured the development of shape changes. By using the same analytical method, ontogenetic shape changes were assessed for human cranium during the fetal period (Morimoto et al., 2008) and for Neanderthals between birth and adults (Gunz et al., 2010). When a large number of samples with distinct groups are available, statistical shape analysis between groups can be performed based on the canonical variate analysis of Procrustes coordinates (Zelditch et al., 2012). Free et al. (2001) used 58 adult MRIs and showed hemispheric differences in shape. Weinberg et al. (2009) reported statistical group difference in shape between MR images with and without orofacial clefting.

The Procrustes analysis performed on the cortical macroanatomical landmarks extracted a principal component that exhibited significant age-dependent change. This change seems to represent a dynamical topological change around the parietal lobe. More specifically, the upper central sulci moved posteriorly and the posterior part of the Sylvian fissure moved anteriorly. As shown in Figure 4B, the variation of the posterior part of the Sylvian fissure was more apparent in the left hemisphere. This indicates that asymmetric development is present around this cortical region. Since the origin is bound to the centroid, and all the points involved were adjusted using scaling and rotation in order to produce the best overlap of the corresponding points in this analysis, the obtained variations should reflect systematic topological changes across the corresponding points. However, a drawback of the Procrustes analysis is that extraction of the topological variations may depend on the initially chosen landmarks. In fact, due to the limited availability of macroanatomical cortical landmarks in the prefrontal regions, the current Procrustes analysis does not reflect topological changes in the prefrontal cortices. The Euclidian distance analysis revealed that the precentral sulci shift anteriorly with respect to the AC and PC as infants develop, suggesting that a proportion of the precentral gyri is enlarged, and this would be detected as a relative posterior shift of the central sulci in the Procrustes analysis.

On the other hand, the Procrustes analysis of 10-20 landmarks on the scalp revealed a distinct principal component with posterior points shifting outward and temporal points shifting inward. These correspond to the relative elongation of the scalp along the anterior-posterior axis and the narrowing of the scalp on both sides. Since Euclidian distance analysis revealed significant elongation of the brain on the y-axis, while changes in brain size on the x-axis for this age range were smaller than those on other axes (Table 1), this principal component is mainly due to growth of the brain and scalp along the anterior-posterior axis.

### Topological relationships among macroanatomical landmarks using euclidian distance

Concerning the general shape of the brain, there was an age-dependent increase in brain size in all directions in both hemispheres. However, the rate of enlargement differed among directions with the brain growing the most rapidly in the anterior-posterior axis, while growth was less eminent in the width and height directions. It should be noted that the evidence of the leftward occipital and rightward frontal asymmetry, known as petalia or Yakovlevian torque (LeMay, 1976; Toga and Thompson, 2003), was not obtained in the present study. This is consistent with the report by Gilmore et al. (2007), which showed that Yakovlevian torque is not observed in neonates. On the other hand, Kabdebon et al. (2014) argued that Yakovlevian torque produces asymmetry of Sylvian fissure in infants of 3–17 weeks of age. Thus, whether such asymmetry of the brain is generated in infants, and at what age, remains an open question.

Concerning macroanatomical structures on the lateral cortical surfaces, there were several obvious changes. The ventral root of the central sulcus, as measured at its imaginary cross fissure, shifted anteriorly in both hemispheres while such change was not observed in the dorsal edge of the central sulci. This change suggests that the ventral area of the frontal lobe moved forward as the brain elongated anteriorly in the course of development. This trend was also supported by the relative anterior shift of two macroanatomical landmarks in the of the ascending rami of the Sylvian fissure and the posterior root of the inferior frontal sulci in both hemispheres. Further, this global change in the frontal area seemed to be associated with a widening of the precentral gyri because the posterior root of the superior frontal sulci moved forward while the dorsal edge of the central sulci remained unmoved as infants developed.

Another macroanatomical change was found in the anterior roots of the intra-parietal sulci shifting anteriorly. Given other observations that relative positions of the parieto-occipital sulci remain rather stable, the anterior shift of the intraparietal sulci may suggest a relative enlargement of the border regions of the superior and inferior parietal lobules in the anterior direction. The other obvious change was the upward shift of the calcarine fissure on both hemispheres. Thus, the occipital lobe also underwent developmental changes. However, changes in the temporal lobe were not as pronounced. The only exception was a relative downward shift of the right inferior temporal sulcus while the left side remained unaffected.

These tendencies in relative changes in macroanatomical landmark positions were mostly in line with the results of macroanatomical delineation performed by Li et al. (2014). The forward shifts of the parietal lobules and the root of the central sulci in the current study were consistent with enlargement of the parietal lobe. This is in line with the previous study on the growth rates of cortical gray matter, showing that higher growth rates were observed in the angular gyri from zero to 2 years of age and in the supramarginal gyri from 1 to 2 years of age (Gilmore et al., 2012). The forward shift of the prefrontal macroanatomical structures including the precentral and inferior frontal gyri found in the current study was also consistent with the widening of these gyri (Li et al., 2014) and increases in the gray matter volume of the gyri (Gilmore et al., 2012). Although the gyrus-oriented approach by Li et al. (2014) and our approach emphasizing major junctions of sulci cannot be compared directly, the two different approaches did not yield any major discrepancies. The development of these regions may be related to language acquisition in the first 2 years, as the development of white matter underlying the left frontal cortex showed significant relationship to expressive and receptive language abilities (O'Muircheartaigh et al., 2014).

Given these analyses, we postulate that macroanatomical landmarks on the lateral cortical surfaces are useful indicators for describing infant brain development. Even with recent advancements in tissue segmentation techniques for low-contrast infant brain images, skull stripping to extract the infant cortical surface is difficult. The infant MRI scans used for the current study did not have sufficient image contrast to undergo automatic tissue segmentation, but were clear enough to allow detection of macroanatomical landmarks defined by orientations of major sulci.

### Analyses of the international 10-10 system

In comparison with approaches using a standard brain template produced based on MRIs, a scalp-based positioning system, such as the international 10-20 system and its derivative the 10-10 system, would serve as a simple and robust approach to providing a common referential framework for the lateral cortical structure, even when the quality of an MRI is not sufficient for skull-striping, segmentation, and normalization. Thus, we explored the relationship between the 10-10 landmarks and macroanatomical cortical landmarks. The cortical landmarks on each infant brain were back-projected to the scalp and expressed in reference to three neighboring 10-10 positions. They were transformed to the scalp template of a 12-month-old infant, who also served as a template for extensive analyses in our former study (Matsui et al., 2014), using three neighboring 10-10 positions as described previously (Okamoto and Dan, 2005). As depicted in Figure 7, variability among macroanatomical cortical landmarks was mostly in the order of several millimeters. In comparison, distances between neighboring 10-10 landmarks were ~23 mm. As we can see, variability in the positions of macroanatomical cortical landmarks was much smaller than the area defined by 10-10 landmarks. This indicates that 10-10 landmarks are sufficient for predicting the underlying macroanatomical cortical structures and that variability in the macroanatomical landmarks, including age-dependent developmental changes, is practically negligible. Thus, we have demonstrated that although the shape of the infant brain undergoes change, relative scalp positioning realized with the international 10-10 system should elastically adjust its shape to maintain a correspondence between scalp and cortical surfaces.

The reliability of the 10-20 system to predict underlying cortical gyral structures has been established for adults (Okamoto et al., 2004) and for infants (Kabdebon et al., 2014). Another line of study indicated that the 10-10 system can separate scalp positions in a non-overlapping manner, but that the 10-5 system is too minute (Jurcak et al., 2007). Although the realm of prediction is limited to the major sulcal junctions, the current study clarifies that the 10-10 system can offer a stable referential framework to predict lateral cortical macroanatomical structures for infant brains.

## Conclusion

The current study demonstrates that macroanatomically distinct cortical structures on the lateral cortical surfaces defined as junctions between major sulci and fissures could serve as useful landmarks for the examination of topological features of brains between birth and 2 years of age. Their detection does not require high-resolution MRIs, and, thus, they serve as robust measures to describe macroanatomical changes in infant brains. A Procrustes analysis detected an age-dependent global trend manifesting as a posterior shift of the upper region around the central sulci and an anterior shift of the region around the posterior edge of the Sylvian fissure. Analyses of relative Euclidean distances among the macroanatomical landmarks revealed general shape differences as well as several distinct regional topological changes, most obvious of which were the forward shift of the macroanatomical structures on the prefrontal cortex and the parietal lobules as infants developed. Importantly, developmental changes in the relative topological orientation of the macroanatomical cortical landmarks were found to be sufficiently smaller than the area defined by the international 10-10 system. Therefore, we propose that the international 10-10 system can serve as a robust referential framework for positional descriptions in fNIRS study on infants.

## Ethics statement

This study was carried out in accordance with the recommendations of the Research and Ethics Committee at the University of Toyama with written informed consent from all subjects.

## Author contributions

ID, FH, GT, and DT wrote the manuscript. MM provided all the primary data and the research environment. DT performed the majority of computational analyses image processing. GT performed the Procrustes analyses. FH and ID performed anatomical analyses. HW arranged research facilities and managed the research schedule. All authors contributed to conceiving research ideas, and interpretation of the results.

### Conflict of interest statement

The authors declare that the research was conducted in the absence of any commercial or financial relationships that could be construed as a potential conflict of interest. The reviewer HM declared a shared affiliation, though no other collaboration, with several of the authors DT, GT, and HW to the handling Editor, who ensured that the process nevertheless met the standards of a fair and objective review.
